# Factors Associated With Late Antiretroviral Therapy Initiation Among People Living With HIV in Southern Iran: A Historical Cohort Study

**DOI:** 10.3389/fpubh.2022.881069

**Published:** 2022-06-17

**Authors:** Sima Afrashteh, Mohammad Fararouei, Haleh Ghaem, Zahra Gheibi

**Affiliations:** ^1^Student Research Committee, Shiraz University of Medical Sciences, Shiraz, Iran; ^2^HIV/AIDS Research Center, Shiraz University of Medical Sciences, Shiraz, Iran; ^3^Department of Epidemiology, Non-Communicable Diseases Research Center, School of Health, Shiraz University of Medical Sciences, Shiraz, Iran; ^4^Department of Epidemiology, Shiraz University of Medical Sciences, Shiraz, Iran

**Keywords:** late antiretroviral therapy, HIV, AIDS, cross-sectional study, Iran

## Abstract

**Objectives:**

Late initiation of antiretroviral therapy (ART) is associated with poor outcome among people living with HIV (PLHIV) and higher risk of transmission of infection. This study was conducted to identify the determinants of late ART initiation among PLHIV in Southern Iran.

**Methods:**

A historical cohort study was conducted on 1,326 PLHIV of ≥15 years of age who were registered with the behavioral diseases counseling center (BDCC) in southern Iran from August 1997 to March 2021. Late ART initiation was defined as a CD4 cell count <200 cells/mm^3^ or having a clinical AIDS diagnosis at the time of ART initiation. The required demographic and clinical data were collected from the patients' medical records. Multiple regression analysis was conducted to define late ART initiation associated factors.

**Results:**

Late ART initiation was found among 81.9% of patients. Based on the results of the multivariate analysis, older age (odds ratio [*OR*] = 1.02, 95% *CI* = 1.00–1.04), being single (*OR*_single/married_ = 1.80, 95% *CI* = 1.17–2.78), history of drug use (*OR*_yes/no_ = 1.64, 95% *CI* = 1.02–2.62), year of ART initiation (*OR*_2011−2013/2018−2021_ = 3.65, 95% *CI* = 2.28–5.86), and possible route of transmission (*OR*_druginjection/sexual_ = 7.34, 95% *CI* = 1.16–46.21) were directly associated with the risk of late ART initiation.

**Conclusions:**

The results show that the prevalence of late ART initiation was alarmingly high. For better infection control and better prognosis of infection, people at high risk need to be provided with timely services (e.g., diagnosis, treatment, training, and social support).

## Introduction

The advent of antiretroviral drugs has reduced HIV transmission and mortality worldwide (1). According to the latest report from the World Health Organization (WHO) in 2020, 37.7 million people are living with HIV (PLHIV) worldwide, of which only 73% have access to antiretroviral drugs (2). One of the major HIV-related challenges around the world is the high rate of late initiation of antiretroviral therapy (ART), which has negative impacts on the quality of life and surveillance of patients and more transmission of the infection (3).

Various factors such as age, gender, low level of education, unemployment, co-morbidities, and injecting drugs have been reported as important predictors of late ART initiation (3–5). Also, social and healthcare systems, stigma of having the disease, the concerns about the side effects of HIV drugs, and psychological problems among patients can affect the timely starting of ART (6–8). Despite the improvements in the availability and costs of ART, there is still a high rate of the late initiation of these drugs in several developing countries including Iran (5, 9, 10).

The current global HIV epidemic is considered as one of the important public health issues. To control this problem, we need early diagnosis of the disease, early onset of treatment, and adherence to the treatment to keep the viral load suppressed in PLHIV (5). Delay in starting antiretroviral therapy is associated with weakened patients' immune function, increased risk of opportunistic infections transmission, increased mortality, increased risk of cardiovascular disease among PLHIV and higher pressure on healthcare systems (5, 11).

Iran has the largest concentrated HIV epidemic among the Middle East countries (12). According to the latest report (2020) from UNAIDS, about 54,000 Iranians are living with HIV and 3,200 PLHIV are dying of AIDS-related illnesses annually. According to this report, the most common way of HIV transmission in Iran is drug injection, and most cases occur in men of 15–44 years of age. Also, only 42% of PLHIV in Iran are aware of their disease status, and only 29% receive ART (12, 13). Despite the importance of HIV and its timely treatment initiation, we found no published information on the rate of late ART initiation among PLHIV in Iran.

Because the late start of ART has many negative consequences and due to the lack of information about the status of treatment of PLHIV in Iran, we decided to investigate the factors associated with late ART initiation among PLHIV in southern Iran.

## Materials and Methods

### Study Design and Data Collection

This is a historical cohort study on PLHIV registered with the behavioral diseases counseling center (BDCC) in southern Iran from August 1997 to March 2021. The center provides health and social care to PLHIV in this part of the country. After the first visit, individuals with HIV-positive diagnosis are to be registered and followed for every 6 months to check for their clinical and ART status. Also, after confirming the diagnosis, all patients are interviewed to complete a structured questionnaire, which included questions regarding demographic characteristics, the status of co-infections, high-risk behaviors, and drug use. In BDCC, trained interviewers collect the required data. In our study, all PLHIV over the age of 15 years who had started ART were included. Exclusion criteria were lack of information about CD4 count at the starting time of the treatment and not starting the treatment. Finally, 1,326 patients were included in the analysis. Based on the ART strategy defined by WHO in 2006, late ART initiation was defined as having a baseline CD4 count <200 or reaching AIDS stage iv before the onset of ART, a definition that is accepted by developing countries at the time and years after (14–16). Patients are diagnosed and treated by a specialized and trained physician in the center.

At the time of diagnosis and registration with BDCC, information regarding the patient's demographic information (i.e., age, gender, level of education, occupation, and incarceration history), high-risk behaviors, history of drug use or drug injection, stage of illness (defined according to WHO by the physician at the first visit), the possible route of disease transmission, CD4 cell count, and tuberculosis co-infection is collected by the trained staffs of the center. The delayed diagnosis was defined as having a CD4 count ≤ 350 cell/mm^3^ at the time of diagnosis (17). As mentioned before, the trained and experienced staffs collect the data *via* a face-to-face interview by completing an official registration form designed by the ministry of health. Informed consent is routinely obtained from the patients or their parents/guardians if they were under 18 years of age at the first visit (*n* = 2).

### Statistical Analysis

Means (*SD*) and percentages were used to report the distribution of continuous and categorized variables, respectively. Chi-square and independent *t*-test were used to measure the association between the grouping and continuous variables in this study, respectively. Also, multiple logistic regression was used to examine the adjusted relationships [odds ratios (*OD*) and 95% *CI*] between the study variables (such as age, gender, education, marital status, occupation, incarceration history, drug use, injection drug use, TB co-infection, diagnosis delay, stage of the disease, route of transmission, and year of ART initiation) and late ART initiation. Variables were included in the model if they significantly contributed to the fitness of the model using the stepwise selection method. The significant cut point was set at *p* ≤ 0.05. Data were analyzed using STATA14.0 software (Stata, College Station, TX, USA).

## Result

**Figure 1:**
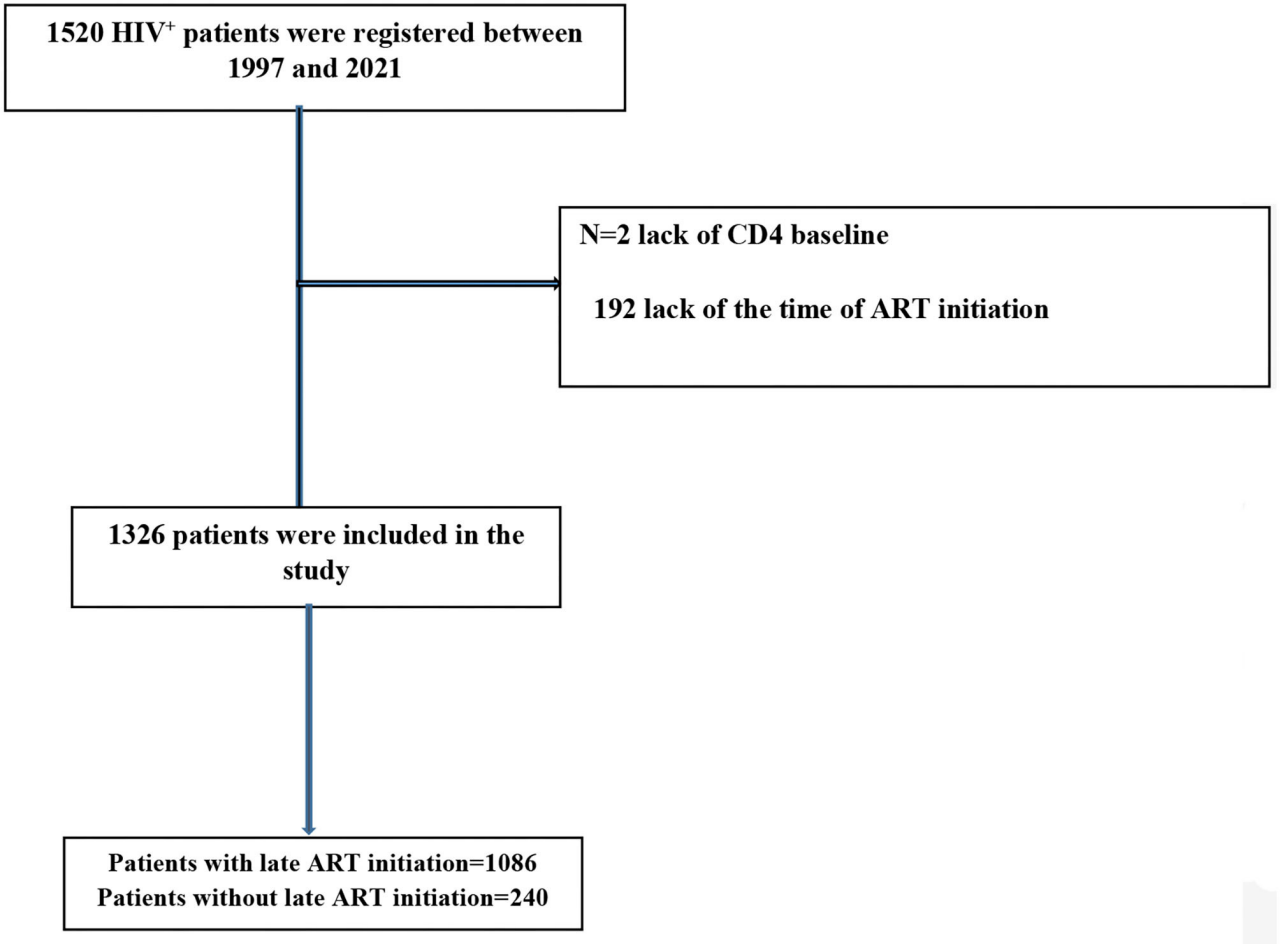
The flow chart of participant's selection.

In this study, the number of patients who were included in the analysis was 1,326 (Figure 1), of which 68.2% were male. The overall late ART initiation rate for the whole period of study was 81.90% (i.e., only 18.09% started ART with CD4 ≥ 200, with 85.4% late ART in 2014–2017 and 65.5% in 2018–2021). The mean age of the participants was ± 45.47 9.49 years, a majority of the participants (88.8%), did not finish compulsory education, and 56.1% were married. Of the patients, 57.6 and 65.3% reported a history of incarceration and drug use, respectively. The most important route of HIV transmission among the patients was drug injection (51.9%). In addition, 431 (32.5%) patients were in stages 3 and 4 of the disease at the time of diagnosis. Several variables, namely, age at diagnosis, gender, marital status, occupation, history of drug use, imprisonment, TB co-infection, delayed diagnosis, stage of the disease, and route of transmission were significantly associated with the late ART initiation (*p* < 0.05). The mean time from the diagnosis of HIV to ART initiation among the late ART group was 33.2 months, four times longer than the non-late ART group (*p* < 0.001). More details are presented in Table 1.

**Table 1:** Analysis of the demographic and clinical data among early and late antiretroviral therapy (ART) initiation patients.

**Characteristics**	**Category**	**Total patients (*N* = 1,326)**	**Patients with late ART initiation (*N* = 1,086)**	**Patients without late ART (*N* = 240)**	***P*-Value**
		**No. (%)**	**No. (%)**	**No. (%)**	
Age group	<30	44 (3.3)	24(54.5)	20 (45.5)	<0.001
	30–39	314 (23.7)	239 (76.1)	75 (23.9)	
	≥40	968 (73.0)	823 (85.0)	145 (15.0)	
Gender	Male	904 (68.2)	780 (86.3)	124 (13.7)	<0.001
	Female	422 (31.8)	306 (72.5)	116 (27.5)	
Education (at diagnosis)	Less than diploma	1,177 (88.8)	971 (82.5)	206 (17.5)	0.115
	Diploma or more	149 (11.2)	115 (77.2)	34 (22.8)	
Marital status	Married	664 (50.1)	524 (78.9)	140 (21.1)	0.003
	Single	315 (23.8)	277 (87.9)	38 (12.1)	
	Widowed/divorced	347 (26.2)	285 (82.1)	62 (17.9)	
Occupation	Employed	608 (45.9)	515 (84.7)	93 (15.3)	0.015
	Unemployed	718 (54.1)	571 (79.5)	147 (20.5)	
Incarceration history (at diagnosis)	Yes	764 (57.6)	666 (87.2)	98 (12.8)	<0.001
	No	562 (42.4)	420 (74.7)	142 (25.3)	
History of addiction	Yes	866 (65.3)	756 (87.3)	110 (12.7)	<0.001
	No	460 (34.7)	330 (71.7)	130 (28.3)	
Injection drug use	Yes	688 (51.9)	609 (88.5)	79 (11.5)	<0.001
	No	638 (48.1)	477 (74.8)	161 (25.2)	
TB/HIV co-infection	Yes	104 (7.8)	93 (89.4)	11 (10.6)	0.045
	No	1,222 (92.2)	993 (81.3)	229 (18.7)	
Diagnosis delay	Yes	967 (72.9)	830 (85.3)	137 (14.2)	<0.001
	No	359 (27.1)	256 (71.3)	103 (28.7)	
Stage of the disease	I, II	895 (67.5)	706 (78.9)	189 (21.1)	<0.001
	III, IV	431 (32.5)	380 (88.2)	51 (11.8)	
Route of transmission	Injection drug use	688 (51.9)	611 (88.8)	77 (11.2)	<0.001
	Sexual route	484 (36.5)	351 (72.5)	133 (27.5)	
	Others*	154 (11.6)	124 (80.4)	30 (19.6)	
Year of ART initiation	Before 2010	232 (17.5)	182 (78.4)	50 (21.6)	<0.001
	2011–2013	374 (28.2)	339 (90.6)	35 (9.4)	
	2014–2017	471 (35.5)	402 (85.4)	69 (14.6)	
	2018–2021	249 (18.8)	163 (65.5)	86 (34.5)	
Time from diagnosis to ART initiation** (months)		1,326 (100)	33.29 ± 39.5	8.7 ± 21.35	<0.001
CD4 cell counts at ART initiation (cell/mm^3^)		1,326 (100)	230.62 ± 1 84.395	351.63 ± 242.65	<0.001

*Other modes of transmission included unknown cause, blood transition, and dentistry.

**Time from the diagnosis of HIV to ART initiation.

Among all study variables (such as age, gender, education, marital status, occupation, incarceration history, drug use, injecting drug use, TB co-infection, diagnosis delay, stage of the disease, route of transmission, and year of ART initiation), the results of multiple logistic regression model provided the adjusted relationships between the selected independent variables (such as age, marital status, drug use, diagnosis delay, route of transmission, and year of ART initiation), and late ART initiation (Table 2). According to the results of multiple logistic regression, older age (*OR* = 1.02, 95% *CI* = 1.00–1.04, *p* = 0.010), being single (*OR*_single/married_ = 1.80, 95% *CI* = 1.17–2.78, *p* = 0.008), drug use (*OR*_yes/no_ = 1.64, 95% *CI* = 1.02–2.62, *p* = 0.038), route of transmission (e.g., *OR*_Injectiondruguse/sexual_ = 7.34, 95% *CI* = 1.16–46.21, *p* = 0.034), diagnosis delay (*OR*_yes/no_ = 2.19, 95% *CI* = 1.57–3.05, *p* < 0.001), and year of ART initiation (*OR*_2011−2013/2018−2021_ = 3.65, 95% *CI* = 2.28–5.86, *p* < 0.001) were significantly associated with the risk of late ART initiation.

**Table 2:** Adjusted association of study variables with late ART initiation 1997–2021.

**Variables**	**Categories**	**Unadjusted** **Odds ratio (95% CI)**	***P*-Value**	**Adjusted** **Odds ratio (95% CI)**	***P*-Value**
Age at the time of diagnosis		1.03 (1.02–1.05)	<0.001	1.02 (1.00–1.04)	0.010
Gender	Male	1	–	1	–
	Female	0.41 (0.31–0.55)	<0.001	Not included	–
Education	< Diploma	1	–	1	–
	≥Diploma	0.71 (0.47–1.08)	0.114	Not included	–
Marital status	Married	1	–	1	–
	Single	1.94 (1.32–2.86)	0.001	1.80 (1.17–2.78)	0.008
	Widowed/divorced	1.22 (0.88–1.71)	0.225	1.18 (0.83–1.69)	0.348
Employment	Employed	1	–	1	–
	Unemployed	0.70 (0.52–0.93)	0.015	Not included	–
Incarceration history	No	1	–	1	–
	Yes	2.29 (1.79–3.05)	<0.001	Not included	–
Route of transmission	Sexual route	1	–	1	–
	Injection drug use	3.01 (2.20–4.10)	<0.001	7.34 (1.16–46.21)	0.034
	Others	1.55 (0.99–2.42)	0.053	1.65 (1.01–2.70)	0.045
History of addiction	No	1	–	1	–
	Yes	2.70 (2.03–3.60)	<0.001	1.64 (1.02–2.62)	0.038
Injection drug use	No	1	–	1	–
	Yes	2.60 (1.93–3.49)	<0.001	0.18 (0.02–1.15)	0.072
Diagnosis delay	No	1	–	1	–
	Yes	2.43 (1.82–3.26)	<0.001	2.19 (1.57–3.05)	<0.001
TB treatment	No	1	–	1	–
	Yes	1.94 (1.02–3.70)	0.041	Not included	–
Year of ART initiation	2018–2021	1	–	1	–
	Before 2010	1.92 (1.27–2.88)	0.002	1.17 (0.74–1.87)	0.485
	2011–2013	5.11 (3.30–7.89)	<0.001	3.65 (2.28–5.86)	<0.001
	2014–2017	3.07 (2.13–4.42)	<0.001	3.06 (2.06–4.54)	<0.001

## Discussion

To our best knowledge, this is the first study on predictors of late ART initiation in PLHIV in Iran. According to the results, the prevalence of late ART initiation in Iran is alarmingly high (81.9%). We also showed that older age, being single, drug injection as the route of transmission and earlier year of ART initiation increase the odds of late ART initiation in PLHIV in Iran.

The prevalence of late ART initiation was 65.5% in 2018–2021. Late ART in Brazil and Ethiopia is reported at about 55.8 and 67.3%, respectively (5, 9). The figure is also much bigger than the rate reported from Canada (48%), Cameroon (35.5%), and Uganda (37.7%) (10, 18, 19). It seems that in Iran, delayed diagnosis of HIV leads to a higher risk of late ART initiation (6, 20).

The present study showed that the older age is one of the risk factors for late ART initiation. A study in Ethiopia, showed that people aged 35–44 years of age were more likely to have late treatment initiation than those aged 15–24 years (9). Studies suggested that due to the lack of knowledge or some ethical issues, older PLHIV are more likely to seek treatment with delay (21, 22). Results of a study in Canada suggested that the delay of starting treatment among older patients may also have more difficulties in accessing care (19). The study emphasizes the urgent need for targeting older people at high-risk to encourage them of seeking HIV testing in order to reduce delay in starting treatment and its negative consequences.

In our study, being single was also significantly associated with an increased odds of late ART initiation. A study in Uganda showed that married people start ART initiation earlier (18). In another study, single PLHIV reported 1.88 times longer delay in starting ART than married people (9). Also, a study in Mozambique found that married and widowed women had a lower risk of late ART initiation than single woman. Married patients may believe that HIV is a possible cause of illness and death of their spouse. This can motivate the person to be diagnosed earlier and seek care for their condition (3). Another possible reason for the late start of treatment in single people is the low family support, which leads to later diagnosis and management of the disease in these patients. However, several studies did not show a significant relationship between marital status and treatment initiation in PLHIV (4, 16, 23).

In the present study, people who use drugs were more likely to experience a delay in the treatment initiation. A study in Canada reported that laws, policies, and programs that address the use of illicit drugs as a public safety issue could positively help public health to address the HIV epidemic (24). O'Neil et al. showed that in a multidisciplinary approach, like a comprehensive case management, consulting services are required to be provided to PLHIV in order to achieve timely initiation and better adherence to treatment (25). In China, an urgent need for early detection of HIV and better access to treatment for injecting drug users is highly suggested (26). Injecting drug users are also a high-profile group for HIV in Iran, and due to the important role of antiretroviral treatments in the reduction of virus load and infection transmission, the focus on the screening methods to identify PLHIV earlier and, therefore; early treatment initiation is of immense importance to prevent spreading infection in the population.

Our results showed that in recent years, the rate of late ART initiation in PLHIV is decreased, indicating a significant improvement in HIV diagnosis and treatment programs. It is necessary to mention that the strategy of ART initiation at the diagnosis for all PLHIV is implemented since 2017 in Iran. Also, diagnosis in groups at high risk and widespread implementation of screening programs for pregnant women are implemented from the beginning of 2017 (before 2017, ART treatment in Iran was based on the amount of CD 4 and disease status) (27). In 2011–2013 and 2014–2017, compared to 2021–2018, the risk of late ART initiation in PLHIV was much higher. This finding has been reported by similar studies (9, 16).

The delayed diagnosis is strongly associated with the late ART initiation in PLHIV. People at high risk for late diagnosis of HIV should be targeted for appropriate interventions to achieve early diagnosis and to be supported to start and adhere to treatment (28). Also, late ART initiation especially in people with delayed diagnosis may contribute to a lower count of CD4 cells (29) and lead to advanced stages of the disease, suppression of the immune system, and a poorer response to ART. For example, in China, several PLHIV who were late diagnosed eventually died even after receiving ART within 1 year of diagnosis (28). These findings highlight the importance of easy, fast, and free access to HIV diagnosis and treatment.

The UNAIDS report suggested that only 29% of patients in Iran receive treatment, so the rate of delay in treatment in Iranian patients is expected to be high (13). To achieve the 90–90–90 goals in Iran, the reasons for missed opportunities for the treatment of PLHIV should be investigated and proper strategies should be applied. Also, removing barriers to access the HIV prevention and treatment services requires appropriate cultural interventions (30). Also, effective policies are needed to strengthen the disease diagnosis system and reduce the diagnosis delay. This will decrease the risk of disease transmission and reduces the number of cases of drug-resistance. The current policy of treating PLHIV in Iran is “treatment for all.” However, delay in the diagnosis of new cases and consequently delay in starting treatment is of the important problems of the HIV control program in Iran (20). Also, in Iran, due to negative perceptions, stigma, discrimination and misinformation about HIV and its treatment, life-long treatment among Iranians living with HIV is undermined (6, 31). In the field of HIV control in Iran, PLHIV needs timely prevention, timely diagnosis, early and proper treatment, long-term care at home, and social and psychological support. Accordingly, health education, socio-economic support, cultural interventions, and condom promotion are recommended for HIV control in Iran. Primary health care (PHC) in Iran can play key roles in supporting groups at high risk with regard to harm reduction, reduction of high-risk sexual behaviors, and diagnosis and treatment of PLHIV to improve the prognosis of HIV infection and reduce transmission (32, 33).

### Strengths and Limitations

In our study, the relatively large sample size provided us with the findings that are to some extent representative of PLHIV in the southern area of Iran. However, having variables with small sample-size categories, a few estimates are noted expectedly precise. Also, the diagnosis rate of PLHIV is low and those who come for diagnosis services are possibly different from those who do not. In addition, although the consultants and interviewers were well-trained to reduce reporting errors in the time of data collection and interview, due to the long follow-up period of patients and some cultural issues including social stigma in Iranian society, errors and bias (especially in reporting drug use, sexual relationships, and incarceration) in our data is not to be ruled out. This is a historical cohort study in which data were collected as a routine procedure during the follow-up period of PLHIV. Again the data collection was fully observed by the research committee of the center and consultants and interviewers were well-trained and experienced. Finally, the observational design of the study prevents us from making any causality inference.

## Conclusion

In the present study, the late ART initiation and its related factors were evaluated. A significant proportion of patients in the present study reported late ART initiation. Late ART initiation may cause the poor response to treatment for patients and higher HIV transmission in the society. Factors such as age, history of drug use, delay in diagnosis, being single, and years of ART initiation indicate a high risk of late ART initiation in PLHIV in Iran. These findings emphasize on the need for early diagnosis and early treatment initiation for people at high-risk. Efforts are also needed to improve care after HIV diagnosis in Iran.

## Data Availability Statement

The data of this study is not publicly available due to its being the intellectual property of Shiraz University of Medical Sciences but is available from the corresponding author on a reasonable request.

## Ethics Statement

The studies involving human participants were reviewed and approved by (IR.SUMS.SCHEANUT.REC.1400.047). The patients/participants provided their written informed consent to participate in this study.

## Author Contributions

SA researched and wrote the manuscript. Also, he contributed to the data collection. HG critically reviewed the manuscript. MF and ZG researched, analyzed, critically reviewed, and edited the manuscript. All authors approve the final version that is submitted.

## Funding

This study was financially supported by the Shiraz University of Medical Sciences, Shiraz, Iran (Grant No. 23264).

## Conflict of Interest

The authors declare that the research was conducted in the absence of any commercial or financial relationships that could be construed as a potential conflict of interest.

## Publisher's Note

All claims expressed in this article are solely those of the authors and do not necessarily represent those of their affiliated organizations, or those of the publisher, the editors and the reviewers. Any product that may be evaluated in this article, or claim that may be made by its manufacturer, is not guaranteed or endorsed by the publisher.
